# Remarks on Mr. Lawrence's Treatment of Corns and Bunyons

**Published:** 1830-10-01

**Authors:** 


### MR. LAWRENCE ON CORNS.

FROM the talented lecturer's short disquisition on corns, we suspect that Mr. Lawrence never suffered from the "pinching of the shoe;" otherwise he would have recommended a more efficacious mode of treatment for this ignoble but very painful complaint. With the etiology, physiology, and pathology of corns, we shall not quarrel. The methodus medendi is not what quadrates with our experience.

"The palliative cure of corns, as we may call it, consists in cutting away the indurated cuticle, so as to remove from the inflamed skin, at all events, this mechanical source of irritation. The feet are first soaked in warm water, so as to moisten the indurated cuticle; you then take a sharp knife and cut away the morbid cuticle which has accumulated over the inflamed parts of the skin. In doing this, after you have removed the inflamed part, you generally come to a sort of point, where the cuticle seems to extend deeper than at the other parts; indeed it appears as if at one point the disease extended farther into the skin than elsewhere, and this has been commonly called the *root* of the corn. It is said that you may lift up and take away in a mass that part of the hardened cuticle which is thus formed in the corn; but I apprehend it is not very easy to do this. *However, if you cut away the thickened cuticle in this manner, and cover the part with soap plaister, or some other mild plaister, spread on leather, and direct the patient to wear shoes that produce no pressure, great relief is experienced.* You find that usually the covering of the cuticle re-forms after this operation; but if the patient avoids the external source of irritation, the corn will not become seriously troublesome. If, however, considerable inconvenience be still experienced, you may proceed to further measures for the more effectual relief of the case—that is, after shaving away all the thickened part of the cuticle—you may rub the skin with lunar caustic. Thus you diminish the inflamed and irritable state of the skin, and then perhaps have no re-formation of the corn, if you avoid the external exciting cause; at all events the patient will derive very great alleviation from this simple process."

We are ready to grant that if the patient, after the erasure of the corn, wears shoes which "produce *no pressure*, great relief is experienced." But where are such shoes to be procured? Certainly not at Hoby's, nor at any fashionable shop in London. If we examine the feet of the Venus de Medicis, the Belvidere Apollo, or even the Farnese Hercules, we can perceive no corns. But modern fashion aims at making the anterior extremity of the foot more narrow than the posterior—the toes more acute than the heel. Mr. Lawrence, therefore, should have recommended, at once, the sandals of the Greeks and Romans, as the only shoes that caused "*no pressure.*" But the gallopades and quadrilles of our times will not harmonize with the sandal; and therefore some remedy must be devised for the "pressure of the times." The simple cover of "soap plaister" is insufficient; and we are much surprized that Mr. Lawrence should have overlooked the obvious measure of throwing the pressure upon less prominent parts, by means of a series of plaisters with holes in their centres, and then one general cover over all. This is the only effectual defence, so long as we wear shoes or boots; and the mechanical explanation of its efficacy does not at all deteriorate from the chirurgical dignity of its etiology.

The Chiropodists of this luxurious age, (for we have now doctors for every part and disease of the body, from water on the brain to horn on the toes) make a great noise about the distinction between corns and bunyons. The following observations of Mr. Lawrence shew that these two affections are often conjoined—or probably stand to each other in the relation of cause and effect.

"There is an affection somewhat allied to, and in fact often actually connected with corns, which, however, in some respects is different from them. It is that kind of swelling which is called *bunyon*. This forms on the prominent joint of the great toe, that is, the joint between the first metatarsal bone and the first bone of the toe, a part of the foot particularly prominent, and thus particularly liable to pressure from the boot or shoe. The swelling thus formed is larger, and generally attended with more redness of the skin and tumefaction, than we see in corn; but besides there is often a hardened and thickened state of the cuticle over the most prominent part of the swelling, which constitutes bunyon. I believe the swelling of bunyon itself consists of inflammation of a bursa mucosa, which is seated between the skin and the prominent part of the joint in question;—a bursa mucosa in a situation which is analogous to that of the patella or olecranon, and by the irritation of a bursa from the pressure of the boot or shoe, a state of inflammation arises with effusion into it. If you open this swelling, you find generally that a fluid escapes. When this is in a state of inflammation, you may adopt the same means that you would do in cases of inflammation of other bursæ — leeches, poultices, lotions, or cold applications. Now the prominent part of the skin is liable to become the seat of corn, and I believe in many instances the irritation thus produced is the cause of the inflammation of the bursa. Sometimes the inflammation of the bursa becomes so considerable that a formation of matter takes place — abscess occurs, and the matter makes its escape externally. If a corn form in this situation, you must adopt the means I have already described to you, and so far as the inflammation itself goes, you have only to adopt the usual antiphlogistic means, and afterwards take measures to protect the part which is the seat of disease from the pressure of the shoe or boot."

